# Queer compromises: How homophobic violence pushed gays and lesbians to make police alliances in Toronto, 1981-2001

**DOI:** 10.1177/13634607251334181

**Published:** 2025-04-17

**Authors:** Andy Holmes

**Affiliations:** 7938 University of Toronto, Canada

**Keywords:** Homophobia, legal cynicism, LGBTQ, police liaison, police brutality, violence

## Abstract

Interactions between LGBTQ2+ communities and the police are historically fraught with violence. LGBTQ2+ activists demand equitable police relationships, legal recognition, and protection under the law. However, LGBTQ2+ groups also tend to hold more antagonistic views of the police. How have gay and lesbian activists navigated this tension? This article draws from 246 archival documents and 313 newspaper articles focusing on events from 1981 to 2001 between gay and lesbian organizations, activists, and the police in Toronto, Canada. This paper illustrates how gay and lesbian activists eventually formed police alliances as a result of homophobic police brutality and public gay bashings. Recognizing the centrality of safety in these conversations, I conclude with a discussion of political compromises LGBTQ2+ investments in policing may take.

## Introduction

Relations between police and gay and lesbian communities have a tumultuous history. Two competing theories explain interactions between police and LGBTQ2+ groups. On the one hand, scholars writing about the demand for law argue gays and lesbians invest in policing for safety (Colvin, 2012; Dwyer, 2014; Russell, 2019). For decades the demand for hate crime protections and the achievement of citizenship have been at the core of LGBTQ2+ politics (Eng, 2010; Jenness and Grattet, 2001; Moran and Skeggs, 2004; Plummer, 2001). However, researchers also recognize many within LGBTQ2+ communities mistrust the police, often across intersectional lines (Grasso et al., 2023; Robinson, 2020). Despite the demand for legal protections, many LGBTQ2+ people view the police as failing to protect them from violence and, therefore, avoid reporting crimes to them (Dwyer et al., 2020; Grasso et al., 2023). According to the framework of legal cynicism, people historically disadvantaged by the law, including racialized and low-income communities, often mistrust the legitimacy of the law and police (Sampson and Bartusch, 1998) despite sometimes partnering with them for safety (Bell, 2016; Campeau et al., 2021; Carr et al., 2007). Provided legal cynicism and the demand for law offer different ways people may approach policing, how have gay and lesbian activists historically transitioned from being victims of police violence to forming alliances with them?

On February 5, 1981, over 200 plainclothes police officers from Division 52 of the Metropolitan Toronto Police in Canada violently raided and arrested approximately 300 men at four gay bathhouses (Warner, 2002). At the time, this was the second-largest mass arrest in Canadian history and a watershed moment for activism against homophobic policing (Warner, 2002). The bathhouse raids were humiliating and represented the nadir of police relations with Toronto’s gay community. Two decades later, Toronto’s first LGBT police liaison committee formed, and uniformed police marched in the city’s Pride parade.^1^ How did this transformation toward amicable police relations develop?

Through a discursive analysis of historical documents in Toronto between the 1980s and early 2000s, this paper argues homophobic police violence and public gay bashings pushed gays and lesbians to form queer liberal (Eng, 2010) investments in policing. This article presents how community safety initiatives included, at first, queer self-defence patrols when police could not be trusted. However, ongoing homophobic violence from the public pushed gay and lesbian activists to develop partnerships with the police in the early 1990s. I discuss how LGBTQ2+ activists may create ambivalent political compromises through desiring safety and citizenship.

## Sexual politics and the law in Canada

Canada’s criminalization of homosexuality began in its formation as a settler-colonial state importing Britain’s anti-sodomy laws (Cannon, 1998). In 1890, the Criminal Code added ‘gross indecency,’ prohibiting sexual actions between two men in public and private with a 5-year prison sentence. In 1892, Canada introduced its first bawdy house law, criminalizing sex work. Later, in 1917, the Canadian Criminal Code was amended to vaguely charge people attending ‘a common bawdy house’ for acts of ‘indecency’ (Warner, 2002). These laws authorized police to raid gay and lesbian bars and bathhouses between the 1950s and the early 2000s^2^ and charge sex workers and adults engaged in sadomasochistic activities (Kinsman and Gentile, 2010).

In 1960, Everett George Klippert was the last person in Canada to be imprisoned for “gross indecency” when he admitted to police to having consensual sex with men (Warner, 2002). When the Supreme Court denied Klippert’s appeal in 1967, it “produced a public outcry” (Warner, 2002: 46). This influenced Prime Minister Pierre Trudeau’s famous proclamation for decriminalizing homosexuality, stating, “there’s no place for the state in the bedrooms of the nation.” That same year, Canada decriminalized sex in private between two men ages 21 and older. However, Section 179 of the Criminal Code continued listing prostitution and “indecent” sexual acts in a common bawdy house as punishable offences (Hooper, 2019). This meant the Attorney General and courts who found gay sex in commercial establishments as vaguely ‘indecent’ could justify police arrests (The Body Politic, 1981a). Despite the decriminalization of homosexuality in 1969, police found ways to charge more than 1300 men in gay bars and bathhouses between 1968 and 2004 under Section 179 of the Criminal code (Hooper, 2019).

Between the late 1970s to 1990s, important legal changes governing sexuality shaped Canadian politics. In 1977, Quebec became the first province to add sexual orientation to its Human Rights Code (Tremblay, 2015). In 1982, Trudeau’s government also introduced the Charter of Rights and Freedoms, providing legal means to legitimize gay and lesbian discrimination cases. By 1986, the Ontario Human Rights Code also added sexual orientation as a prohibited ground for discrimination, followed by the province of Manitoba and the territory of Yukon in 1987 (Tremblay, 2015). Over the summer of 1991, Toronto City Council officially recognized “Pride Day” after a decade of refusing to do so. In 1995, the Supreme Court then ruled in *Egan and Nesbitt v. Canada* that sexual orientation was analogous to other protected identities under the Canadian Charter of Rights and Freedoms (Smith, 2005). By 1996, the federal Canadian Human Rights Act and Canadian Criminal Code were amended to consider sexual orientation when sentencing crimes motivated by hate (Tremblay, 2015). Laws like these validated gay and lesbian citizenship by showing little tolerance for homophobic violence (Jenness and Grattet, 2001).

## Toronto’s gay neighbourhood and activism

These legal changes allowed for burgeoning gay culture and activism in the late 1960s and early 70s, making Toronto “Canada’s ‘homosexual capital’” (Nash, 2005:116). Between the late 1960s and 80s, Toronto’s gay neighbourhood emerged around two intersecting streets, Church and Wellesley, inhabited predominately by white gay men (Nash, 2005; Ross, 1995). From the 1960s through the 70s, gay-owned businesses in Toronto including bathhouses offered spaces for men to safely meet like-minded people (Bruner, 1981; Hooper, 2016). These bathhouses included The Barracks, which opened in 1974, and The Club, Roman II Health, and the Richmond Street Health Emporium (Hooper, 2016).

Activism continued from the mid-1970s to 80s. In 1976, the formation of the Lesbian Organization of Toronto (LOOT) offered avenues for women’s political organizing distinct from gay men’s groups (Ross, 1995). By the 1980s, several social movement groups in Toronto within the gay and lesbian movement emerged, including Black feminist lesbians who formed the Toronto Black Women’s Collective (Bain, 2017). Similarly, in the 1980s, Zami, Toronto’s first group for Black lesbians and gays, and Gay Asians Toronto formed to support racialized members underrepresented in mainstream, white and middle-class gay organizations (Warner, 2002).

During the 1960s, some gay men broadly held assimilationist values prescient of queer liberal goals (Eng, 2010; Nash, 2005). Assimilationist groups, like the University of Toronto Homophile Association in 1969, framed gay men as inherently similar to heterosexuals to achieve full integration into mainstream society (Nash, 2005; Warner, 2002). These goals contrasted significantly with the emergence of gay liberationists with radical demands for sexual freedom (Ferguson, 2019). Notably, in 1971 the publication of *The Body Politic*, an influential revolutionary magazine based in Toronto, represented gay liberationist ideology covering topics of sexual freedom. In contrast to assimilationists, gay liberationists wanted to free sexuality from heteronormative constraints and viewed sexuality as fluid and unfixed (Nash, 2005; Warner, 2002). The variations in these gay and lesbian goals preface broader changes in the aftermath of Toronto’s bathhouse raids and the eventual shift towards joining forces with the police.

### The demand for law and police

Criminologists, sociologists, and cultural scholars argue the pursuit of citizenship is a strong reason why LGBTQ2+ groups invest in greater legal protections, including the policing of hate crimes (Grattet and Jenness, 2008; Moran and Skeggs, 2004; Plummer 2001). Hate-motivated violence directed at particular social groups became recognized in the 1980s and 1990s to deter criminal acts based on hatred (Jenness and Grattet, 2001). It is well-established that lesbian, gay, bisexual, and transgender people are disproportionately targeted by hate crimes (Bender and Lauritsen, 2021; Katz-wise and Hyde, 2012; Statistics Canada, 2024). Yet, in Canada federal police-reported hate crime data, including on sexual orientation, was not collected until 2005 (Dauvergne et al., 2006). Nonetheless, between 1990 and 2004, there were 121 community-reported cases of people killed because they were known or suspected to be gay, lesbian, bisexual or transgender (36 were in Toronto) (Douglas, 2005).

One way gays and lesbians advocate for law and safety is through LGBTQ2+ police liaison programs (Colvin, 2012; Dwyer, 2014). Police liaisons attempt to strengthen relationships between officers and communities historically targeted by violence (Stasiulis, 1989). However, this can be challenging for LGBTQ2+ groups if police organizations have homophobic and masculine cultures (Burke 1992; Cherney, 1999). For instance, lesbian and gay officers often emphasize physically aggressive traits to counter the perception of their emasculated identities in a heteronormative occupation (Miller et al., 2003). By the late 1990s, police organizations nevertheless began embracing “diversity politics” in the workforce, creating opportunities to improve relations with gays and lesbians. Research shows police organizations with hate crime policies and closer ties with the communities they interact with increase the likeliness of people targeted by hate crimes to report them (Grattet and Jenness, 2008). While research on LGBTQ2+ liaison officers is relatively new, scholars discuss their emergence to alleviate hostile relations between queer communities in places like Australia in the 1980s (Dwyer et al., 2020), and Washington D.C. between 2000 and 2001 (Colvin, 2012). Although research has evaluated the efficacy of LGBTQ2+ police liaison officer programs (Dwyer et al., 2020), this paper provides a detailed description of events leading up to the formation of an LGBTQ2+ police liaison committee in Toronto in 2001.

### Skepticism of police

While more likely to be victims of hate crimes, LGBTQ2+ individuals are also more likely to mistrust the police (Dario et al., 2020; Grasso et al., 2023; Vogler and Jenness, 2023). This varies by gender and race where in the United States Black and Hispanic LGBTQ2+ people are less likely to report violence to police, and 72.6% of transgender and 96.7% of gender non-binary people view the police negatively (Vogler and Jenness, 2023). One reason for this could be because of homophobic police cultures. Scholars writing about police occupational culture (Bowling et al., 2019; Loftus, 2010) argue that police officers often hold homophobic views that negatively shape their interactions with gays and lesbians (Cherney, 1999). For instance, despite Canada decriminalizing homosexuality in ‘private’ in 1969, between 1990 and 2004 there were 37 recorded cases of police raids, arrests, or intimidation in queer spaces, eight of which were in Toronto (Douglas, 2005). This matters because negative perceptions of police among lesbian, gay, bisexual and queer people deter their reporting of crimes (Grasso et al., 2023).

For LGBTQ2+ people of colour, it can be further challenging to trust the police when experiencing racialized forms of police harassment (Meyer, 2015; Robinson, 2020). This relates to how the liberal inclusion of gays and lesbians in politics often dissociates sexuality from racial inequalities (Eng, 2010; Ferguson, 2019). Queer liberalism describes the desire for sexual minorities to be recognized as citizens under the laws of the state (Eng, 2010). Reminiscent of homonormative desires to be accepted in mainstream politics (Duggan, 2002), according to David Eng under queer liberalism, “the state emerges as the central locus and guarantor through which non-normative sexual identities it once criminalized are now protected” (2010: 28).

Criticism of queer liberal investments in policing can be traced to anti-racist and working-class groups that emerged during gay and lesbian liberation (Duberman 2018). After the 1969 Stonewall riots, activists forming the Gay Liberation Front (GLF) in New York supported black civil rights, protested the Vietnam War, and challenged capitalist inequalities (Duberman, 1993; Ferguson, 2019). Similarly in the 1960s and 1970s, intersectional feminists saw little liberation in white, middle-upper-class feminists striving for workplace equality by exploiting racialized, lower-class women (Collins, 2000; Lorde, 1984). However, these critical views were not shared among liberal and politically moderate members of New York’s GLF. As historian Martin Duberman argues, some activists wanted “to win acceptance for gays within the country’s institutional structure – not to topple or transform that structure” (1993: 232). Reforming systems (including law enforcement) to include gays and lesbians rather than radically restructuring them is a cornerstone of queer liberal politics. Therefore, people skeptical of law enforcement may surprisingly seek better policing, believing police should exercise their authority equally – not unfairly (Campeau et al., 2021; Carr et al., 2007). By analyzing important moments of homophobic violence in Toronto, this paper argues both skepticism and the demand for law catalyzed queer liberal investments in policing.

## Data and methods

This paper analyzes three archival sources: Digitized material from *The Body Politic*, digitized material from *DailyXtra*, and the ArQuives, one of the world’s largest independent LGBTQ2+ archives located in Toronto (Sheffield, 2020). (See Table 1). *The Body Politic* was Canada’s first radical gay liberation magazine published monthly between 1971 and 1987. *DailyXtra* (rebranded as *Xtra Magazine*) began in 1984 shortly before *The Body Politic* discontinued, and since 1999 has been publicly available online. While gay men wrote the majority of articles in *The Body Politic* (exceptions include lesbian writers like Chris Bearchell), lesbian feminists often published in *Broadside: A Feminist Review* between 1979 and 1989. Because this paper draws upon a wide corpus of material from *The Body Politic*, it focuses (though not exclusively) on the experiences of gay men.

**Table 1.** Documents analyzed.

Physical archive	Folder names	Pages analyzed
ArQuives	Brief from RTPC + Metro Toronto Police commission and “OUR POLICE FORCE TOO 1979-Jun 1981. Folder no. F0141-04-050	51
ArQuives	CGRO brief to Arnold Bruner 1981. Folder no. F0044-04-039	4
ArQuives	Correspondence between Metro Toronto Police chief and RTPC 1981-1982. Folder no. F0141-02-008	6
ArQuives	Gay/Police relations analysis, newspaper clippings, TBP issue #76, 1981-1983. Folder no. F0002-04-250	1
ArQuives	Letter from RTPC Acting chair to Toronto Star 1981. Folder no. F0141-02-005	1
ArQuives	Metro coalition for police reform (no Folder no.)	18
ArQuives	Minority-police relations. Folder no. F0044-04-107	71
ArQuives	Police-community relations symposium social action committee, Ontario Association of professional social workers. 1982-1983. Folder no. F0141-04-085	14
ArQuives	Police-gay community relations conference 1980. Folder no. F0044-05-005	2
ArQuives	Police-gay relations; correspondence, reports, press clippings, draft articles, 1979-1982. Folder no. F0002-03-157	21
ArQuives	Toronto LGBT community police liaison committee 2000-2001. Folder no. F0129-002-064	24
ArQuives	Toronto mayor’s committee on community and race relations. Sub-committee on lesbian and gay issues in Toronto. Folder no. 55G2	26
ArQuives	“We are a community” RTPC brief to City’s neighbourhoods Committee: Re: Bruner’s report and gay/Police relations reports 1981. Folder no. F0141-04-054	2
ArQuives	Unlabelled folder. Folder no. M2013-151	4
ArQuives	Unlabelled folder (no Folder no.)	1

Newspaper	Years analyzed	Articles
DailyXtra	(1999-2005)	247
The body politic	(1981-1987)	66

Doing a discourse analysis of documents involves understanding how complex social issues are framed for the public (Fairclough, 2003). As historian Arlette Farge argues, archival documents show unexpected “lives of ordinary people who would otherwise be ignored” (2013:1). Historical research offers perspectives over time, while interviews are limited to those still alive. However, qualitative interviews with people in Toronto would have yielded rich in-depth experiences with the police otherwise difficult to capture from merely anecdotal depictions of events from archival sources. Nonetheless, newspapers, though secondary sources, can corroborate first-hand written archival data, particularly when analyzing historically underrepresented communities. This is because knowledge production is narrative, interpretive and determined by what people value remembering and preserving (Stoler, 2002; Trouillot, 1995). Laws that criminalized homosexuality and the distribution of ‘obscene materials’ in Canada between the 1970s and early 2000s likely shaped what documents were deemed possible to safely preserve.

I accessed digitized records from 1981 to 1987 of *The Body Politic* through the website of the Canadian Museum for Human Rights. This period covers Toronto’s 1981 bathhouse raids to the last year of publication of *The Body* Politic. My search term was “violence,” which yielded 66 articles online. For *DailyXtra*, I included 2005 as the upper bound of my search to capture 25 years after the bathhouse raids and the first year police Chief Bill Blair marched in Toronto’s Pride parade, marking a significant change in police-LGBTQ2+ relations. My search term was “police” which produced 247 *DailyXtra* articles. My research at the ArQuives started with their online catalogue and the search term “police”. Archivists helped me access 15 folders relevant to this project and I photographed 864 archival items, in which I analyzed 246 of them. I copied these archival documents into NVivo where I analyzed them for qualitative themes first with an initial round of open coding followed by analytic coding (Auerbach and Silverstein, 2003). In total, I generated 87 open-coded themes which I amalgamated into the categories of “homophobic police violence,” “homophobic violence from the public,” and “police liaison committees.”

## Findings

The findings show a chronology of how queer liberal investments in policing developed in Toronto between the early 1980s and early 2000s. This development was shaped by homophobic violence from both the police and the public, and the emergence of Toronto’s first LGBT police liaison committee. Each section articulates how violence pushed gays and lesbians to work with the police to gain safety.

### Homophobic violence from police

On December 9^th^, 1978 Toronto police raided and charged 28 men at the Barracks, a bathhouse well-loved among leather and S&M clientele (Lee et al., 1979). In response, activists in Toronto quickly developed the Right to Privacy Committee (RTPC) to provide legal aid for men the police arrested. The RTPC submitted a formal report titled “Our Police Force too! A Brief Presented on Behalf of the Toronto Gay Community” (Lee et al., 1979) to the Metropolitan Toronto Board on April 5^th^, 1979. This document was co-authored by a professor at the University of Toronto, John Alan Lee, alongside activists Paul Trollope, Peter Maloney, Kathy Orlita, George Hislop, and Brent Hawkes. Of notable interest was the RTPC’s recommendation “That the Police Department appoint an officer, of the rank of Sgt. or above, as a full-time liaison between the Police Dept. and the Lesbian/Gay community” (Lee et al., 1979: 25). This was among the first times in Toronto members of the gay and lesbian community asked for a police liaison.^3^

Despite the RTPC advocating for better dialogue with police, on February 5, 1981, at 11 p.m., over 200 undercover plainclothes officers from Division 52 of the Metropolitan Toronto Police violently raided four gay bathhouses. Police arrested 309 men and laid 286 charges while inflicting an estimated $35,000 in property damage to the bathhouses (Hannon, 1981a). One cashier at a raided venue, the Richmond Street Health Emporium, recalled police telling men arrested in a shower room, “Too bad the showers aren’t hooked up to gas,” a homophobic remark alluding to the Holocaust (The Body Politic 1981b: 8). Police later released the names of those arrested at the bathhouses, whose names were sent to their employers and families to destroy their reputations. This was a form of non-consensual outing historically promoted by homophobic police cultures in Canada (Kinsman and Gentile, 2010).

The bathhouse raids in 1978 and 1981 pushed Toronto City Council to alleviate hostile police behaviour. On July 13, 1981, after a vote of 18-3, Toronto City Council allocated $22,500 to commission a report on issues between police and Toronto’s gay and lesbian community (Jackson, 1981: 7). Consequently, Arnold Bruner, a 54-year-old articling law student and former journalist at the time, agreed to create this report. Bruner’s report (1981) provided a rare opportunity for gay and lesbian activists to inform municipal policies on police relations. For instance, on August 14, 1981, a group called Gay Fathers of Toronto wrote to Bruner urging police to reconcile relationships with gay men (Gay Fathers of Toronto, 1981). Their letter framed themselves as law-abiding citizens despite the Canadian state casting them as deviant:

Like the vast majority of Canadians, we want our children to grow up in a society that adheres to the rule of law, with a police force that even-handedly enforces the law. We were brought up to respect the police, to see the police as our protectors. And we want our children to follow our example. But in addition to being fathers, we are also gay, and when a man acknowledges this, he also realizes that he can no longer count on the policeman as his ally and friend…he hears reports of homosexuals being ridiculed at police functions; worst of all, he has to live in a grey area where the line between what is legal and what is illegal is imprecise.

Existing “in a grey area” of legal jurisdiction, Gay Fathers of Toronto sought to alleviate hostile relations with police. The 1981 Bathhouse raids lead Gay Fathers of Toronto to urge Bruner to recommend the police recruit gay and lesbian officers. They stated, “In recent years, women and members of Toronto’s ethnic communities have been recruited to the police force and we urge a similar recruitment of gay men and women as a way of improving relations with our community” (Gay Fathers of Toronto, 1981). In fact, in 1976 Toronto police officers and Black communities formed the Liaison Group on Law Enforcement and Race Relations (Stasiulis 1989). By the late 1970s gay and lesbian activists in Toronto began framing themselves as an analogous group to ethnic communities (Nash, 2005). Nonetheless, in their letter to Bruner, Gay Fathers of Toronto expressed their concern about the police:

Unfortunately, gay men are now so suspicious of the police that a visit by a couple of officers at any gay event is interpreted as a sign of an impending raid. Are we being utopian to hope that, in the future, relations between the police and gays would be such that a policeman on his beat could pay a friendly visit to a gay bar and be welcomed?

This fear of impending raids depicts an early moment when gay activists were not just cynical of police, but also hopeful of improving interactions with them for safety.

Bruner’s report also drew the attention of civil rights groups including the Coalition for Gay Rights in Ontario (CGRO). Unlike Gay Fathers of Toronto, the CGRO took a more critical approach to the police. In their report sent to Bruner on August 18, 1981, the CGRO scrutinized attacks on gay rights:

…the Government and the police are working hand in hand in a carefully orchestrated, politically motivated campaign directed against the gay and lesbian communities. The Government, through the Attorney General, is allowing, indeed encouraging, the police to harass and intimidate gays. The police are assisting the Government in promoting an anti-gay backlash that will prevent the inclusion of “sexual orientation” in the Human Rights Code (Coalition for Gay Rights in Ontario, 1981).

While the Ontario Human Rights Code eventually added sexual orientation as prohibited grounds for discrimination in 1986, police and conservative politicians attempted to thwart this legislation. In their sardonic criticism, the CGRO condemned Ontario’s Solicitor and Attorney General, Roy McMurtry, and the Chief of Toronto’s police, stating, “Roy McMurtry and Jack Ackroyd have jumped into bed together but it is the gay community that is getting screwed.” Hence, the CGRO urged Bruner to consider in his report:

Whatever problems exist between our community and the police department will not go away by writing some mildly worded report that attempts to soft pedal the issue…You must use the opportunity you have to convince the police department and the Ontario Government to change their tactics…You must tell the Attorney General and the Chief of Police that the gay community is not the problem. They are the problem.

As the CGRO argued, Bruner’s report should demonstrate the legitimacy of the gay and lesbian community to police and for police to recognize their homophobia.

While Bruner’s report made 16 recommendations to the Metropolitan Police (Bruner, 1981), only two were approved by Toronto City Council on November 19, 1981 (Metropolitan Toronto Board, 1982). These accepted recommendations can be traced to the activist work of groups like Gay Fathers of Toronto and the CGRO. The first recommendation involved the Chief of Police, Jack Ackroyd, clarifying to staff that “the gay community constitutes a legitimate community with Metropolitan Toronto…entitled to the same respect, service and protection as all law-abiding citizens” (Bruner, 1981:176). The second approved recommendation included the establishment of a “Gay Awareness Program” (Bruner, 1981:178) to educate police on gay and lesbian issues. These two recommendations were among the first formal requests by Toronto city councillors that police ameliorate hostile relationships with gay and lesbian communities. However, the language of “law-abiding citizens” should be scrutinized, considering sex between men in ‘public bawdy houses’ continued to be interpreted by police as grounds for prosecution under Section 179 of the Criminal Code (Hooper 2019). How could police respect “law-abiding citizens” if gay sex was being targeted for ‘public safety’? Bruner’s report nonetheless was a turning point as it humanized Toronto’s gay and lesbian community and negatively framed the police as homophobic (Hooper, 2016). But it was the work of activist groups like the RTPC, Gay Fathers of Toronto, and the CGRO that vocalized concerns of unjust police violence.

### Homophobic violence from the public

**Figure 1.**
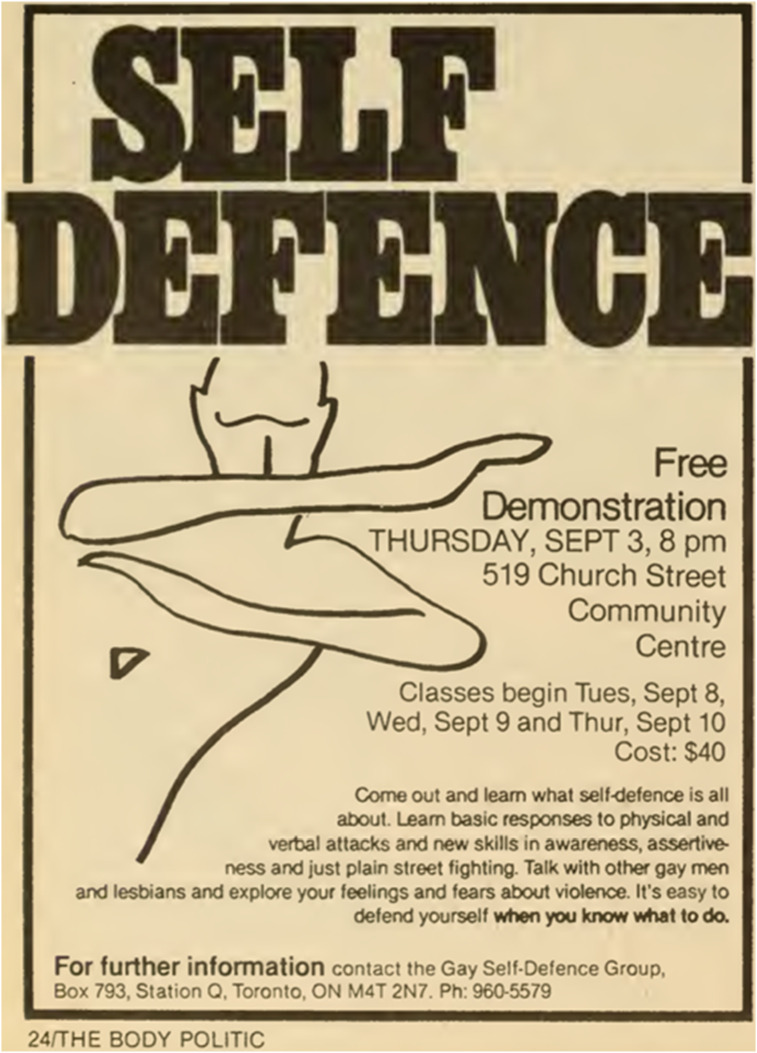
Anti-homophobia self defence poster.

Gays and lesbians encounter homophobic violence not only from the police but also from the public. Fear of gay bashers further contributed to forging police relationships for public safety. Following the February 1981 bathhouse raids, people feared police would condone homophobic attacks. Posters distributed throughout Toronto’s gay and lesbian communities like one advertised in *The Body Politic* (The Body Politic 1981c) encouraged people to protect themselves through self-defence classes (see Figure 1).

On May 14, 1981, Toronto’s largest gay and lesbian organization, the RTPC, announced the creation of a street patrol service called Toronto Gay Patrol (TGP) (Popert, 1981). According to *The Body Politic*, the TGP formed “to compensate for inadequate police protection and to embarrass police into taking action against queer-bashing incidents” (Cockerline, 1982: 10). Like previous research on gay street patrols (Hanhardt, 2008) the TGP taught people how to avoid and address homophobic violence, with members patrolling popular downtown streets on Friday and Saturday nights. Advertisements appearing in *The Body Politic* in May 1981 called for participation in an “RTPC-sponsored group trained to patrol streets and lanes where frequent queerbashing occurs” (The Body Politic, 1981d: 35). These advertisements addressed concerns of not only homophobic police but also violent strangers.

Gays, lesbians and feminist groups created ad-hoc street patrols because police could not be relied on (Valverde and Cirak, 2003). The 1981 July/August edition of *The Body Politic* stated, “Many gays are afraid to go to the police; they don’t want to be on record as gay persons in police reports…many gays have concluded that protection from queerbashing can come only from the gay community itself” (Popert, 1981: 11). Additional to gay men mistrusting the police, in 1974 when police failed to take the concerns of women experiencing sexual assault in Toronto,^4^ women formed the Toronto Rape Crisis Centre (TRCC) (Doe 2003). However, in 1986, women from the TRCC and Women Against Violence Against Women (WAVAW) faced resistance from Toronto police to implement training on sexual assault cases because of “pre-existing experiences and prejudices…and the inherent sexism of police culture” (Doe, 2003: 57).

Because legal cynicism and the risk of prosecution curtailed dialogue between gays and lesbians with the police, queer people yearned alternative modes of safety. Since the late 1960s, drag queens, gay, lesbian, and bisexual people celebrated Halloween at the St. Charles Tavern, but thousands of youth would annually throw eggs at attendees chanting “kill the queers” (Hannon, 1981b). What should have been a haven for queer joy became a place of homophobic attacks and the inadequacy of police to reliably intervene. While police in Toronto previously arrested those chanting “kill the queers” in 1979 and 1980, in 1981 (the same year as the bathhouse raids) “there were no arrests” (Hannon 1981b: 11). Queer activists therefore were forced to protect themselves, with their only options being themselves, the TGP and the police. In the September 1982 issue of *The Body Politic*, upon multiple gay bashings in Allan Gardens, activists including Doug Chin from the Tri-Aid Charitable Foundation contacted “police chief Jack Ackroyd complaining about the lack of uniformed police in the park” (Bartley, 1982: 14). This investment in policing coincided with the disbanding of the TGP in the summer of 1984 when fewer people participated in the program (Popert, 1984). Requesting uniformed police in gay bashing locations represented a significant departure from police avoidance. Police were beginning to be seen as protectors rather than just perpetrators of violence.

**Figure 2.**
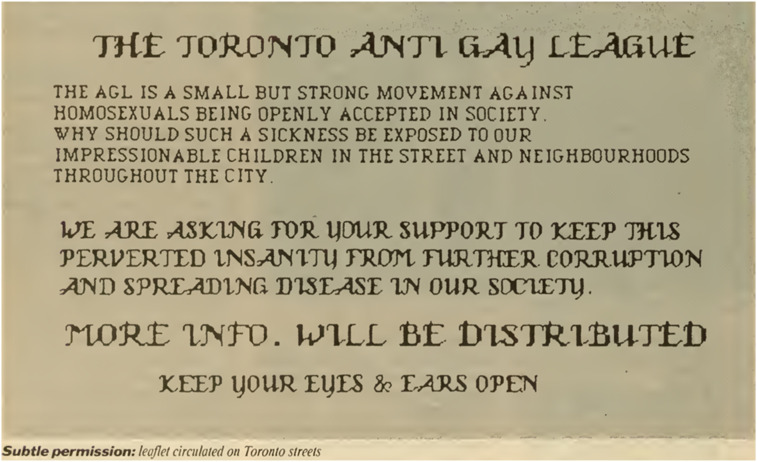
The Toronto Anti gay league.

Yet, gay bashings continued. One horrendous example involved Kenn Zeller, a 40-year-old gay man, teacher, and former President of Toronto’s Teacher-Librarian Association. Five teenage boys killed Zeller in Toronto’s High Park after he left a colleague’s party on June 21, 1985. According to coverage in *The Body Politic*, his killers declared earlier that night: “Let’s get money from a queer!” and “Let’s beat up a fag!” (Lesk et al., 1986: 13). Before Zeller was attacked, one of the boys screamed at him, “You fucking faggot!” before charging at him (Lesk et al., 1986: 13). As noted earlier, hate crimes based on sexual orientation were not recognized in Canada’s Criminal Code until 1996. As Zeller ran towards his car to reach his keys, the teenage boys repeatedly bashed his head, demanded money, and eventually killed him. Zeller’s cause of death reported in *The Body Politic* was “severe cranial cerebral injuries” (Lesk et al., 1986: 15). In reporting Zeller’s death, *The Body Politic* also commented on a leaflet dropped by gay bashers in Toronto in August 1985 (Lesk et al., 1986: 14). This leaflet promoted the Toronto Anti Gay (sic) League (see Figure 2), an organization suppressing the public existence of gays and lesbians.

Zeller’s death prompted policy changes. In 1986 (the same year Ontario added sexual orientation to its Human Rights Code), School Trustee Olivia Chow recommended Toronto’s School Board (as Zeller was a teacher) prohibit discrimination based on sexual orientation and to implement this into the educational curriculum (McCaskell, 1986). Across incidents of homophobic violence was a demand for greater legal and police protection despite police being arbiters of homophobic violence.

### Seeking police protection

Following the slow implementation of recommendations from the Bruner report and the disbanding of the Gay Patrol, police and gay and lesbian communities were at a standstill. There was little publicly noted change until 1991, 6 years after Zeller’s murder. On May 16, 1991, at The 519 Community Centre, discussions of police in gay and lesbian spaces continued at a recruitment meeting for members of the Lesbian and Gay Issues Committee (LGIC) (Visser, 1991a). This committee was chaired by Harvey Brownstone, a defence lawyer for victims of Toronto’s 1981 raids, who believed in the possibility of forming alliances between police and gay and lesbian communities. *DailyXtra* reported he “acknowledged that increasing the visibility of the police in places that gays frequent may make some people unhappy” (Visser, 1991a). He also stated “increasing foot patrols for protection will probably result in more arrests for public sex” (Visser, 1991a). For some gay men seeking safety, this was a minor compromise to make to prevent persistent homophobic violence. Brownstone continued, “I think gaybashers should be on notice that their actions are an offence that won’t be tolerated anymore…it’s going to take enforcement, prosecution and a commitment on the part of the courts if we’re going to deal with this kind of hate-motivated crime” (Visser, 1991a). Brownstone’s investment in law reflect gay and lesbian social movements in Canada leveraging“liberal rights-claiming” discourses of litigation as activism (Smith, 2005: 347). Like with queer liberal politics (Eng, 2010), gay activists in Toronto prioritized seeking human rights laws (Nash, 2005).

To illustrate, on November 18, 1991, the City of Toronto organized the first LGIC meeting at City Hall. Initially, the LGIC was a sub-committee of the Mayor’s Committee on Community and Race Relations. However, City Council recommended this Race Relations committee address the growing need “to promote tolerance and acceptance” of gays and lesbians (Toronto Mayor’s Committee, 1991). As reported by *DailyXtra* in this meeting, “police relations with the lesbian and gay communities was the major topic of discussion” (Visser, 1991b). Eight members attended, including Harvey Brownstone, and police representative, Constable Jim Sneep. This meeting involved stories of gay bashings, a mandatory 90-min sensitivity training course for all officers of the Metropolitan Police, and discussing the presence of police in gay and lesbian spaces (Visser, 1991b). While the bathhouse raids shattered any trust gay men had in the police, Brownstone publicly stated, “you can’t dismiss people’s concerns, but at the same time we can’t live in the past. I think the police are legitimately concerned about gay-bashing” (Visser, 1991a). While Brownstone realized working with police to address gay bashings was “a very complicated issue” he also stated “I think if one has to balance the interest of safety with other interests, then safety would have to be the priority” (Visser, 1991a). This compromise is indicative of the ambivalence of mistrusting police while turning to them for protection.

Although government hate-crime data on sexual orientation was not collected in the early 1990s, according to The 519 Community Centre, data from a ‘gay bashing hotline’ between 1991 and 1993 recorded over 300 incidents (Dembo and Millard, 1993). To address this violence, starting on June 21, 1993, activists joined forces with Toronto’s Metropolitan Police to create a 30-s advertisement on local television warning gay bashings could result in criminal prosecution. This was the first time a city in North America aired anti-homophobia public service announcements on prime-time television (Dembo and Millard, 1993). Simultaneously, between June 15 to July 11, 1993, the LGIC collaborated with the Metropolitan Toronto Police to launch a public awareness poster campaign on gay bashings. Posters designed by the City of Toronto reminded passersby that “Being Gay is Not a Crime. Bashing is” (Mayor’s Committee on Lesbian and Gay Issues, 1993). Both television and poster advertisements represented an important turning point: Police could no longer be seen as only perpetrators of homophobic violence if they now offered protection from it.

This shift was seen through The 519 Community Centre’s establishment of the Victim Assistance Program. According to a letter written to Toronto gay and lesbian community groups in January, 1995 by Karen Baldwin, The 519 Victim Assistance Program and Educational Trainer, this program developed strategies for gays and lesbians to best “respond to and protect ourselves from hate crimes,” support “information and advocacy for lesbians and gay men who have experienced verbal harassment, a bashing or domestic violence” and “to provide training to the police on anti-lesbian and gay violence” (Baldwin, 1995). This program developed growing relations between police and gay and lesbian needs for safety.

In 2000, the Victim Assistance Program then initiated a call for gay and lesbian representatives for “the formation of a queer community police liaison structure in Toronto” (Toronto Police Liaison Plan, 2000). Considering between 1995 and 2000 the Victim Assistance Program developed amicable connections with 17 divisions of Toronto’s police force (Toronto Police Liaison Plan, 2000), it is unsurprising they wanted a formal gay and lesbian police liaison committee. Between June and September 2000, The Police Liaison Working Group, an organization of volunteers, established a formal LGBT Police Liaison Committee (LGBT Police Liaison Working Group, 2001). Three meetings were held in 2000 on January 3, March 8, and June 7. During these meetings, 200 LGBT groups in Toronto were contacted, with 39 sending representatives to at least one of the meetings. Themes from these meetings included concerns about the “police crackdown of sex in the parks,” a “lack of sensitivity of police officers to domestic violence in same-sex relationships, concern about police recognition of hate crimes,” “the community’s trust in police,” concerns for “economic viability” of businesses in Toronto’s gay-village, and the surprising “possible role of the TPS [Toronto Police Services] as role models against hate, homophobia, and heterosexism” (Toronto Police Liaison Plan, 2000). These meetings culminated in the City of Toronto’s first formal police gay and lesbian liaison committee in May 2001 (June 13 Committee et al., 2001).^5^

However, not everyone was eligible to apply for the LGBT police liaison committee or agreed to its role in subverting homophobic violence. On May 30, 2001, activists in Toronto including Maggie’s Toronto Prostitute’s Community Service Project, and the Coalition for Lesbian and Gay Rights in Ontario strongly opposed the formation of the LGBT Community Police Liaison Committee. They argued, “there is no consensus in our community that such a committee can be a useful way to communicate with the police” (June 13 Committee et al., 2001). They elaborated:

Sex trade workers, the women charged in connection with the Women’s Bathhouse, people charged with offences related to the medical use of marijuana, bathhouse found-ins, and political activists could all face exclusion. While the liaison group claims to oppose this restriction, and to have encouraged prostitutes to seek election, it has NOT resolved this issue BEFORE having an election, exposing community members to public embarrassment if they are blocked from joining the committee (June 13 Committee et al., 2001).

Critics of Toronto’s police liaison committee’s eligibility requirements highlighted its exclusion of groups facing continual police violence. For instance, on Sept 14, 2000, six male police officers raided an all-female lesbian bathhouse at the Pussy Palace, forcing attendees to strip searches (Hammers, 2008). This LGBT police liaison committee discouraged people with a history of criminal charges from applying, posing a problem for anyone charged during police raids of LGBT spaces despite the last one in Canada happening in 2004 (Douglas, 2005). The exclusionary criteria suggested only a select type of “queer” as worthy of working amicably with the police – respectable and law-abiding citizens (Duggan, 2002; Eng, 2010).

Given the initial exclusionary criteria of this LGBT police liaison committee, it is unsurprising that gay political goals seeking citizenship status have seldom allied with sex workers and racialized queer rights (McKenna, 2022; Ross and Sullivan, 2012). In Toronto, during the same time LGBT police liaison committees organized, intersectional work around women’s safety was being organized by activists like Beverly Bain, a Black lesbian woman, who experienced resistance from the Toronto Police Services Board (TPSB). Between 1998 and 2000, lobbyists for women’s safety in anti-violence cases led City Council to establish a Sexual Assault Audit Steering Committee in 2005 in collaboration with the TPSB (Bain, 2010). However, by 2007 the TPSB prematurely ended this committee, determining such a group was unnecessary (Bain 2010). While some gays and lesbians successfully could create a police liaison committee in Toronto, this was not universally possible.

## Discussion and conclusions

LGBTQ2+ groups have demanded legal protections for decades (Eng, 2010; Jenness and Grattet, 2001; Moran and Skeggs, 2004; Nash, 2005) and collaborated with police for safety (Dwyer, 2014; Russell, 2019). However, many queer people continue to hold adversarial views of the police (Grasso et al., 2023; Vogler and Jenness, 2023), and avoid reporting crimes to them (Dwyer et al., 2020). This paper contributes a historical chronology over two decades about how gays and lesbians transitioned from holding antagonistic relations with the police towards developing amicable partnerships. Gays and lesbians originally could not turn to police for protection from gay bashings on the streets because of homophobic police brutality. They instead formed community-led safety initiatives such as the Toronto Gay Patrol which provided at first informal queer forms of protection from hate crime violence. However, when this program stopped, activists sought safety with the police and the development of Toronto’s first LGBT police liaison committee.

This paper contributes to how both legal cynicism and the demand for police protection develop with ambivalence when people mistrust the police but rely on them for safety (Bell, 2016; Carr et al., 2007). For instance, Gay Fathers of Toronto’s (1981) plea to be recognized as citizens by police coincided with their mistrust of them. Similarly, in 1991 when the LGIC desired police foot patrols in gay neighbourhoods to prevent gay bashings, this was a compromise made for safety. Today, straddling between safety and insecurity can foster feelings of ambivalence for LGBTQ2+ people (Ghaziani and Holmes, 2023), especially within contexts of violence (Moran and Skeggs, 2004) and police reform (Russell, 2019).

An important implication of queer liberal investments in law enforcement is how sexuality rights become enmeshed with racialized, and class politics of domination (Eng, 2010). This includes restricting migration by framing racialized immigrants as a harbinger of homophobic hate crimes (Haritaworn, 2010), to white gay men gentrifying neighbourhoods with sex workers in the 1970s and 80s in Vancouver and Toronto (McKenna, 2022; Ross and Sullivan, 2012). LGBTQ2+ activists therefore often make sacrifices and political compromises in the name of safety. Some may see assimilatory politics toward the law and policing as presenting a kind of ‘progressive annihilation’ where activists are absorbed into institutions sustaining punitive inequalities (Bernstein, 2010; Puar, 2007). This assimilation may overshadow the anti-racist and class-consciousness politics of early queer organizing (Duberman, 2018; Ferguson, 2019; Nash, 2005). However, gay and lesbian activism in Toronto between the early 1980s and early 2000s demonstrates what may appear to be assimilatory behaviour must be analyzed under the context of violence and the requisite need for safety.

Understanding these historical relations contribute to understanding contemporary conflicts between LGBTQ2+ communities and the police. Currently, police are banned from participating in Pride events globally due to the mistrust some LGBTQ2+ people have in the police (Holmes, 2021; Vogler and Jenness, 2023). For instance, in 2017 Toronto was the first city to introduce a police ban in Pride parades following Black Lives Matter protesting their 2016 presence. In 2018, police were still barred from Toronto’s parade due to concerns about how they investigated the disappearances of immigrant, racialized, and working-class men from Toronto’s Gay Village by a white gay serial killer (Holmes, 2022). Hence, LGBTQ2+ people overall continue to mistrust police more than non-LGBTQ2+ people (Dario et al., 2020; Owen et al., 2018). This is especially the case among transgender, gender non-binary, and racialized LGBTQ2+ individuals (Vogler and Jenness, 2023). Ongoing conflicts between the police and members of LGBTQ2+ communities demonstrate the importance of understanding the history of violence and the need for safety that shapes these challenging relationships.

## Notes

1. Changing relations with the police coincided with broader acceptance towards gays and lesbians in Canadian society. By 2000, most Canadians saw same-sex relations as tolerable (Andersen and Fetner, 2008).

2. Between the late 1960s and 1980s, the Royal Canadian Mounted Police (RCMP) also spied on gay and lesbian feminists labelled a national security threat for affiliating with socialist movements during the Cold War (Kinsman and Gentile, 2010).

3. The first known gay and lesbian police liaison in Canada was Sergeant Bill Cathcart in 1977 in Ottawa McLeod DW (2016).

4. In 1987, a woman by the name of Jane Doe sued the Toronto Police Force (and later won in 1998) citing negligence in their duties to protect her and women from a serial rapist known as the “Balcony Rapist”.

5. The first openly lesbian and gay police officers in Toronto were Laura Rowe and Constable Brian Aguiar in 1991. By 2001 Judith Nosworthy, a lesbian officer, was appointed as Toronto’s “first full-time officer to the gay and lesbian community” (Warner, 2002: 294).
